# Relationship Between Self-Concept, Self-Efficacy, and Subjective Well-Being of Native and Migrant Adolescents

**DOI:** 10.3389/fpsyg.2020.620782

**Published:** 2021-01-27

**Authors:** Cristian Céspedes, Andrés Rubio, Ferran Viñas, Sara Malo Cerrato, Eliseo Lara-Órdenes, Javier Ríos

**Affiliations:** ^1^Facultad de Administración y Economía, Universidad de Santiago de Chile, Santiago, Chile; ^2^Facultad de Economía y Negocios, Universidad Andres Bello, Santiago, Chile; ^3^Facultad de Psicología, Universidad Diego Portales, Santiago, Chile; ^4^Facultat d’Educació i Psicologia, Universitat de Girona, Girona, Spain; ^5^Facultad de Educación y Ciencias Sociales, Universidad Andres Bello, Talcahuano, Chile; ^6^Facultad de Psicología y Psicopedagogía, Pontificia Universidad Católica Argentina, Buenos Aires, Argentina

**Keywords:** self-concept, self-efficacy, subjective well-being, migration, adolescence

## Abstract

In the last decade, the migrant population in Chile has substantially increased, where the rates have not only increased in the adult population, but also among children and adolescents, creating a potential for social and cultural development in the educational system. The present work analyzes the relationship between self-concept, self-efficacy, and subjective well-being in native and migrant adolescents in Santiago de Chile. The sample consisted of 406 students, 56.65% women, with an age range that fluctuated between 12 and 16 years, with an average of 13.36 years (*SD* = 0.96). Student’s *t*-tests were used to compare the average of the constructs evaluated between natives/migrants and boys/girls participants. Subsequently, two multivariate models of simple mediation were constructed, one for natives and another for migrants, which assumed subjective well-being as a dependent variable, academic self-concept as an independent variable and the general self-efficacy as a mediating variable. In both models, gender was considered as a control variable. Results show that migrant students present higher levels of academic self-concept and general self-efficacy than native students. There are no differences with regard to well-being. In the case of gender, differences are observed only for the case of general self-efficacy, where boys present higher levels. On the other hand, a partial mediation is observed for the model of native students and a total mediation for the model of migrant students. The study yielded interesting results regarding the differences in the evaluation of the constructs of self-concept, self-efficacy, and subjective well-being in both groups. Such data can be used as inputs for the development of public policies for adolescents.

## Introduction

Chile has seen in recent years, nearly a million migrants enter its borders, coming mainly from Latin America and the Caribbean, where factors such as political stability, security levels, and constant economic growth throughout the last decades, have turned Chile into a pole of attraction for people seeking better employment and development opportunities (Godoy, 2019). This human movement has brought along thousands of children and adolescents, who have been integrated into the Chilean school system, representing a significant number of municipal school enrollment in the districts with the highest index of habitability of immigrants. In this way, it can be observed that in Santiago, the capital of the country, there was a significant percentage increase in foreign students enrolled in public schools in just 3 years, where it went from 8.9% of foreign students to 15.5% in 2017 (Ministerio de Educación, Centro de Estudios, 2018), modifying the cultural and ethnic composition in the classrooms.

In relation to studies on migrant adolescents in contexts of South–South movements, that is, massive displacement of people from developing countries to others in the same condition but with better economic and human development indices, Chile constitutes a case and studies by Alvites and Jiménez (2011) and Tijoux (2013) begin to shed light on the problems experienced by migrant students due to challenges that the stress of acculturation process implies (Berry, 1997; Mera-Lemp et al., 2020). The immigrant paradox theory suggests that this movement may have an effect in school performance (Suárez-Orozco et al., 2009) and the persistent gender differences in detriment of girls (Alfaro et al.,2016) in the Latin American context may also have consequences in different psychological constructs.

In the case of young foreigners who now live in a country other than their own, their behavior may be shaped by the sum of environmental factors, behavioral and personal aspects, in direct interaction with the degree of stress involved in moving to a geographical place that is not the place of origin, with new customs and values in order to adapt to the new reality of a different community (Berry, 1997; Prilleltensky and Prilleltensky, 2007). In the case of Chile, migrant adolescents have evidenced depressions, anxiety and nostalgia regarding the place of origin (Villacieros, 2020) which may play against the necessary emotional and sociological resetting required to adapt into a new society (Mera-Lemp et al., 2020).

The stage of human adolescence, whether in natives or migrants, is traditionally considered as conflictive, and includes questions and difficulties inherent to its evolution and gender (Oliva et al., 2010). Among the psychological resources related with a good psychological adjustment and social integration of adolescent in the school experience the following constructs can be found: self-concept, self-efficacy, and subjective well-being (Pajares, 1996; Martínez-Antón et al., 2007; Jiménez and Hidalgo, 2016).

Self-concept, widely studied from its multidimensionality (Shavelson et al., 1976; Valentine et al., 2004), since, as indicated by Shavelson et al. (1976) this construct of self-perception is the result of interaction and experience with others on levels such as the academic, emotional, and social, among others. In this sense, it can be assumed that the school experience in a new sociocultural and educational setting puts into play the self-concept (Goffman and Guinsberg, 1970) of migrant adolescents, since as studies carried out in Spain show, students who presented low socialization and self-concept obtained a low academic performance (León del Barco et al., 2007; Plangger, 2015). In the case of the adolescent population, academic self-concept is one of the most relevant personal characteristics when it comes to explaining, for instance, subjective well-being (Huebner, 1991). The studies by McCullough et al. (2000) concluded that academic self-concept was the main predictor of well-being and that measuring it was a good way to understand the well-being of adolescents. The previous point suggests that the adolescent’s self-concept will play a fundamental role in self-assessment, as well as with respect to psychological well-being and the affirmation of one’s own identity (Harter, 1998; Luján, 2002). To the best of our knowledge, there are currently no studies in Chile measuring academic self-concept indices neither in migrant adolescents nor in natives. However, it is a relevant issue since authors such as Hay et al. (1998) affirm that high self-concept is positively related to performance, integration and relationships in the school context, while it is negatively correlated with anxiety. In terms of gender differences on academic self-concept, the study by Costa and Tabernero (2012) did not launch statistically significant gender differences, however, studies by Padilla Carmona et al. (2010) showed that girls surpasses boys in terms of academic self-concept. In Chile, studies such the one of Gálvez-Nieto et al. (2017) did not evidence statically significant differences. Following this same line, it can be considered that one of the most relevant challenges faced by migrant adolescents is the adaptation to a school setting different from that of their country of origin, where self-efficacy, understood as the capacity perceived by an individual to successfully face situations of daily life (Bandura, 1986), this construct plays a crucial role in the inclusion and interaction of individuals, in this case migrants, who join the new group (Briones et al., 2005). According to Briones et al. (2005), self-efficacy in the experience of migrant adolescents suggests a very positive aspect between the level of self-efficacy and the degree of satisfaction with the achievement obtained. Likewise, students who report higher levels of social self-efficacy also notice a greater degree of comfort in environments aimed at sociocultural interaction and better skills in the field of integration. Studies such as that of Juárez-Centeno (2018), indicate that migrant families with a low level of self-efficacy experience higher levels of depression, which affects the behavior of adolescents. Studies carried out in Colombia such as that of Gómez-Garzón (2018), with people who are victims of forced displacement; suggest that there would be a positive relationship between self-efficacy and other constructs such as belonging, inclusion and social well-being. The study of Fan and Mak (1998) in Australia, found that migrant students show lower level of self-efficacy compared with natives as well. In Chile, however, there is still no comparative research in adolescents on self-efficacy of local residents compared to migrants. In terms of gender, contributions made by Blanco Vega et al. (2012), in the Latin American context, suggest that boys tend to have greater indexes of self-efficacy. Similar results were obtained by Junge and Dretzke (1995) and Huang (2013). Quality of life, an important motivational factor in migratory processes, has been conceptualized and measured in different ways. One of them is the concept of subjective well-being, which is positioned within the hedonic tradition and serves as an approach to the satisfaction and happiness of individuals with their own life (Diener, 1994; Cummins and Cahill, 2000). In the context of migration, studies suggest that factors such as time of residence, legal status, size of the social network and coverage of basic needs (Basabe et al., 2009) would be positively related to subjective well-being while Factors such as discrimination (Murillo and Molero, 2012) would be negatively related. Along the same lines, studies such as that of Panzeri (2018) point out the importance of post-migration subjective well-being as a valid measure related to future labor productivity, mental health and social integration from the otherness and needs of the migrants themselves. Regarding the perception of subjective well-being among the native population and the migrant population, Bilbao et al. (2007), Herrero et al. (2012), Muñoz and Alonso (2016), and Hendriks and Bartram (2019) have found that migrants they tend to exhibit lower levels. Similar results have been found in Chile in studies carried out by Alfaro et al. (2016) regarding adolescents. In this regard, gender differences in favor of men in the Ibero-American context could lead to the assumption that the migration process in girls could have a negative effect in terms of some psychological constructs. This is evidenced by studies by Oyanedel et al. (2015) and Alfaro et al. (2016) in Chile, where boys have higher scores on this scale. Similar results were obtained in studies in Spain such as that of González-Carrasco et al. (2017) where it is concluded that the homeostatic system of girls is probably more sensitive to external variations and that there is a relationship between physical and cognitive aspects that occur in girls as well as their specific pattern of subjective well-being.

In this regard, studies on subjective well-being in the adolescent population have been carried out under cross-cultural formulations such as that of Casas et al. (2015) with adolescents from Latin-speaking countries (Spain, Romania, Brazil, and Chile), with adolescents from the two Latin American countries having the lowest scores in terms of subjective well-being. However, studies comparing subjective well-being in migrants and locals are still needed to provide key information to different actors and thus guide decision-making in preponderant sectors, positioning well-being as the center of attention in the development of public policies and as part of the strategies to improve the quality of life (Lucas and Diener, 2008).

Now, regarding to the constructs of self-concept, self-efficacy and subjective well-being. Literature has reported relations among them (García-Fernández et al., 2016). For example, the three of them operate at the level of self-perceptions in social, emotional, and behavioral terms (Bandura, 1992; Lent et al., 1997; Casas et al., 2007). Similarly, these three constructs are sensitive to the context and are built or modified according to lived experiences and social interactions, so they are not stable but rather dynamic (Bandura, 1986; Shavelson and Marsh, 1986). In the educational context, self-efficacy is linked to confidence since students evaluate this ability in order to solve problems, while self-concept is related to the perceived personal competence when executing a task (Bong and Skaalvik, 2003), both constructs emerge as personal competencies in adolescence, which serve as positive development indicators (Oliva et al., 2010). In the case of subjective well-being, this construct is positively related with self-concept and self-efficacy in the educational setting (Gómez et al., 2007; Reyes-Jarquín and Hernández-Pozo, 2011; Malo et al., 2011; Chavarría and Barra, 2014).

**FIGURE 1:**
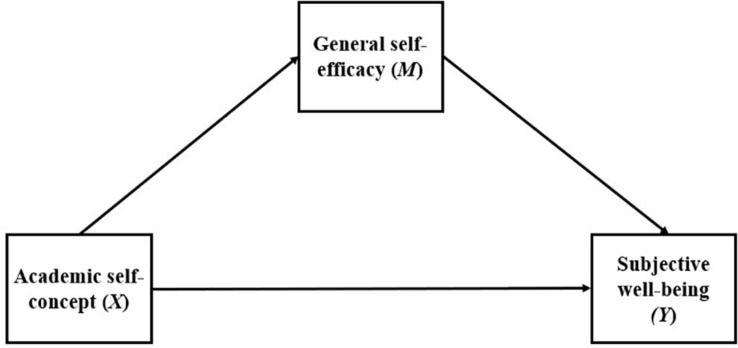
Mediational Model: effect of academic self-concept on subjective well-being through general self-efficacy.

As explained, various studies show a positive correlation between subjective well-being and other variables such as self-concept, and self-efficacy (Huebner, 1991; Harter, 1998; McCullough et al., 2000; Luján, 2002; Gutiérrez and Gonçalves, 2013). However, there is still a lack of studies of this nature in Chile. In the same line, literature has reported that self-efficacy would play a mediational role between subjective well-being and other constructs such as meaning in life, life satisfaction and even personality traits such as extraversion and openness (Krok and Gerymski, 2019). To the best of our knowledge the relations between self-concept and subjective well-being have been widely described but no contribution has been made regarding the mediational role of self-efficacy between these two constructs (see Figure 1). For this purpose, the following conceptual mediational model of self-efficacy in the relationship of between self-concept and subjective well-being has been proposed.

Considering the information above, it can be noted that in the Chile’s school context, there is a gap in the literature regarding the differences between migrant and native students regarding the relationship between self-concept, self-efficacy, and subjective well-being. One might think that there would be an effect on the part of the levels of self-concept and self-efficacy on the subjective well-being exhibited by these students, but there are indications to affirm how this relationship between natives and migrants could be differentiated. Facing a new educational context, adapting to new models, new relationships and especially to a whole new society could put to the test all the cognitive and affective areas that would influence the global satisfaction (Caprara et al., 2006) of migrant students, hence a different configuration of the relationships between self-concept, self-efficacy, and subjective well-being for them (with respect to the natives) could happen not only due to the condition of local or native but because of the gender. In this respect, works by Oyanedel et al. (2015) and Alfaro et al. (2016) in Chile evidenced gender differences in terms of subjective well-being and satisfaction with life with male scoring better in these constructs. Similar records were obtained by González-Carrasco et al. (2017) in Spain finding statistically significant differences. However, studies comparing migrant adolescents with locals in terms of subjective well-being as well as self-concept, self-efficacy and gender, to the best of our knowledge, are not available.

In this new scenario, where Chile is a recipient of migrants, this study aims to constitute a contribution in the investigation of relationships on academic self-concept and self-efficacy regarding the subjective well-being of migrant adolescents versus the local population, including also gender differences. The importance lies in the fact that both groups will coexist to form part of the productive and intellectual assets of Chile, where a clear understanding of behavioral aspects of these groups would guide the efforts of central and local governments in improving public policies for the optimal personal development of all the country’s adolescents. In the same line. This research may serve as input to improve school experience for both locals and migrant students.

This research has set as objective to compare the levels of self-efficacy, academic self-concept and subjective well-being among migrant adolescents and local adolescents. This research also aims at exploring gender differences. On the other hand, the objective is also to analyze how the variables of general self-efficacy and academic self-concept are related, as well as to observe their effect on subjective well-being for each of the study subsamples (migrant and local adolescents).

## Materials and Methods

### Sample

The present study is quantitative and considered a cross-sectional design. The sample was made up of adolescent students belonging to 7th and 8th grade of the Chilean educational system. The students correspond to four municipal public educational centers located in the district of Santiago, Metropolitan Region of Chile. Regarding the selection of schools, two criteria prevailed: convenience and percentage of migrant enrollment (not less than 20%).

As a criterion to calculate the sample size, a 5% error was considered, with a confidence level of 95%. The sample is made up of 406 students, distributed evenly among the four participating establishments. 56.65% of the students were women and 43.35% were men, 45.81% were in 7th grade and 54.19% were in 8th grade, while the age fluctuated between 12 and 16 years, with an average of 13.36 years (*SD* = 0.96). Regarding the key variable of the study (native or migrant condition), the sample consisted of 55.91% of students born in Chile and 44.09% of migrants with similar percentages of school vulnerability index. 28.09% of the migrant students were from Peru, 21.35% from Venezuela, 18.54% from Colombia and 32.02 from other Latin American countries and the rest of the world. The average residence time of migrant students in Chile was 2.59 years (*SD* = 1.68). For the purposes of this study, Chilean-born students were considered as Chilean and foreign students with at least 1 year and a maximum of 5 years in Chile as migrants.

### Instruments

#### AF5 Academic Self-Concept

The AF5 scale (García and Musitu, 1999) emerges as an improved version of the AFA Scale (Form A Self-Concept). The AF5 Scale has been developed taking self-concept as a multidimensional construct based on the works of Shavelson and Marsh (1986). The AF5 Scale was validated in Chile by Riquelme Mella and Riquelme Bravo (2011), showing validity and internal consistency. Under these conditions, the present study showed a high internal consistency (Cronbach’s Alpha of 0.824).

#### General Self-Efficacy Scale

The General Self-efficacy Scale is an instrument developed by Schwarzer and Jerusalem (1995) and measures individual perception in relation to the abilities to cope with daily situations in stressful circumstances. In Chile, Cid et al. (2010) demonstrated internal consistency or homogeneity when obtaining a high Cronbach’s alpha coefficient, similar to the results obtained in other Spanish-speaking countries. In this study, the observed Cronbach’s alpha was 0.859.

#### Personal Well-Being Index – School Children 7 (PWI-SC7)

The Personal Well-Being Index (PWI) was developed and validated by Cummins and Lau (2005), its validity and reliability being demonstrated. Later, there was an adaptation of this instrument to apply it in populations of children and adolescents, generating the PWI-SC 7 Scale, which is a version of seven questions that has been validated in Chile by Alfaro et al. (2013). This instrument uses 11-point scales for responses, ranging from Strongly Disagree (0) to Strongly Agree (10). In the different applications carried out in Chile, the instrument has shown a good factorial fit (one dimension), observing in this study a high internal consistency, with a Cronbach’s alpha value of 0.864.

In addition to these variables, participants were also asked about the different sociodemographic variables: age (in years), sex (0 = boy; 1 = girl), country of birth, time they have been in Chile if they were not born in this country and parents’ country of birth.

### Procedure

The self-report questionnaire was applied to the students, after having obtained the corresponding permits from the directors of the educational centers and subsequent authorization to be taken at agreed times. On the other hand, an informed consent form was given to the students and their tutors. The application was developed in regular school hours of adolescents, during the 2018 school period (within the months of August–November). The material was delivered to the students, the instructions were given and then the time they needed to respond was allowed. In each application a responsible teacher and one or more researchers were present in the classroom.

Schools in the Metropolitan Region of Chile were contacted for convenience, considering the criteria established for the study (having at least 20% migrant students). The only exclusion criterion was that of handling the Spanish language: students from non-Spanish-speaking countries, who had not yet managed the Spanish language to understand the instructions and content of the applied instrument, were left out.

### Analysis of Data

First, descriptive analyzes were performed for the total scores of each scale (for the total sample, and the natives/migrants and boys/girls subsamples). Subsequently, *t*-tests were performed for independent samples for each of these variables, with the aim of comparing the mean scores obtained for each subsamble. Subsequently, two multivariate models of simple mediation were constructed (Hayes, 2018), one for the subsample of natives and another for the sub-sample of migrants, which assumed subjective well-being of the students as a dependent variable (PWI-SC7), academic self-concept as an independent variable and general self-efficacy as a mediating variable. In both models, gender (0 = boys; 1 = girls) was considered as a control variable. ABCa bootstrapped CI based on 5,000 samples was used to calculate the confidence intervals of all the models used.

The statistical analyses were carried out through IBM-SPSS v.24 and the modeling tool PROCESS for SPSS v2.10 (Hayes, 2018).

## Results

### Descriptive and Comparative Results

Table 1 presents the mean and standard deviation of the scores obtained on the academic self-concept, general self-efficacy, and subjective well-being scale, for the total sample and the sub-samples of migrants and natives. In addition, Independent Samples *t*-test are also presented to establish differences between these groups. For the three comparisons, equality of variances was assumed, based on Levene’s test. The differences were statistically significant (*p*-value < 0.05) for the case of academic self-concept and general self-efficacy (being the subgroup of migrants the one that obtained the highest score). No statistically significant differences were observed for these groups in the case of subjective well- being.

**TABLE 1:** Descriptive results and comparisons of means of variables by country of birth.

	Mean (*SD*)			Levene’s Test		Indepentent Samples *T*-Test		
			
	Total (*N* = 406)	Natives (*n* = 227)	Migrants (n = 179)	*F*	*p*-value	*t*	*df*	*p*-value
Academic self-concept	6.14 (2.03)	5.94 (2.05)	6.40 (1.98)	0.19	0.66	–2.31	404	< 0.05
General self-efficacy	2.91 (0.63)	2.85 (0.64)	2.99 (0.61)	0.71	0.40	–2.29	404	< 0.05
Subjective well-being	7.68 (1.89)	7.75 (1.91)	7.59 (1.86)	0.06	0.81	0.86	404	0.39

Table 2 presents the mean and standard deviation of the scores obtained on the academic self-concept, general self-efficacy, and subjective well-being scale, for the total sample and the sub-samples of girls and boys. In addition, Independent Samples *t*-test are also presented to establish differences between these groups. Equality of variances was assumed for the case of general self-efficacy and subjective well-being, while different variances were assumed for the case of academic self-concept (based on Levene’s test). The differences between the mean scores were statistically significant (*p*-value < 0.05) only for the case of general self-efficacy, where boys had higher scores than girls.

**TABLE 2:** Descriptive results and comparisons of means of variables by gender.

	Mean (*SD*)			Levene’s Test		Indepentent Samples *T*-Test		
			
	Total (*N* = 406)	Boys (*n* = 176)	Girls (*n* = 230)	*F*	*p*-value	*t*	*df*	*p*-value
Academic self-concept	6.14 (2.03)	6.22 (1.87)	6.08 (2.15)	3.95	< 0.05	0.72	397.02	0.47
General self-efficacy	2.91 (0.63)	2.99 (0.65)	2.85 (0.60)	1.11	0.29	2.20	404	< 0.05
Subjective well-being	7.68 (1.89)	7.86 (1.80)	7.54 (1.95)	2.15	0.14	1.72	404	0.08

### Mediation Analyzes

The results of the simple mediation models are presented below, which considered subjective well-being as a dependent variable, academic self-concept as an independent variable, general self-efficacy as a mediator variable, and gender as a control variable. Model 1 considered the subsample of natives, while model 2 considered the subsample of migrants.

**FIGURE 2:**
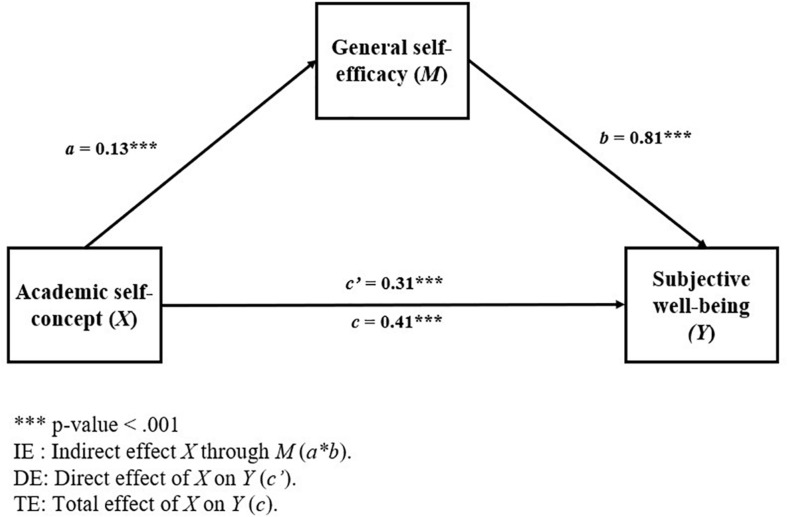
Mediational Model 1: effect of academic self-concept on subjective well-being through general self-efficacy, controlling for gender (subsample: natives).

The results of the regression analyzes that make up the mediation model 1 are presented in Table 3, while the general diagram of this model is presented in Figure 2.

**TABLE 3:** Linear regression analyzes for Mediational Model 1 (sublample: natives).

	Consequent							
	
*n* = 227		*M* (General self-efficacy)				*Y* (Subjective well-being)		
				
Antecedent		Coeff.	*SE*	*p*		Coeff.	*SE*	*p*
*X* (Academic self-concept)	*a*	0.13	0.02	< 0.001	*c’*	0.31	0.06	< 0.001
*M*(General self-efficacy)		−	−	−	*b*	0.81	0.19	< 0.001
Gender		–0.14	0.08	0.08		–0.11	0.23	0.62
Constant	*i*_*M*_	2.18	0.13	< 0.001	*i*_*M*_	10.28	0.65	< 0.001

		*R*^2^ = 0.18				*R*^2^ = 0.26		
		*F*(2,224) = 24.23, *p* < 0.001				*F*(3,223) = 26.40, *p* < 0.001		

A partial mediation can be observed in this model, where the total effect (TE: *b* = 0.41, 95% BCa CI [0.30, 0.52]), the direct effect (DE: *b* = 0.31, 95% BCa CI [0.20, 0.43]), and the indirect effect (IE: *b* = 0.10, 95% BCa CI [0.05, 0.17]) of academic self-concept on subjective well-being were statistically significant.

**FIGURE 3:**
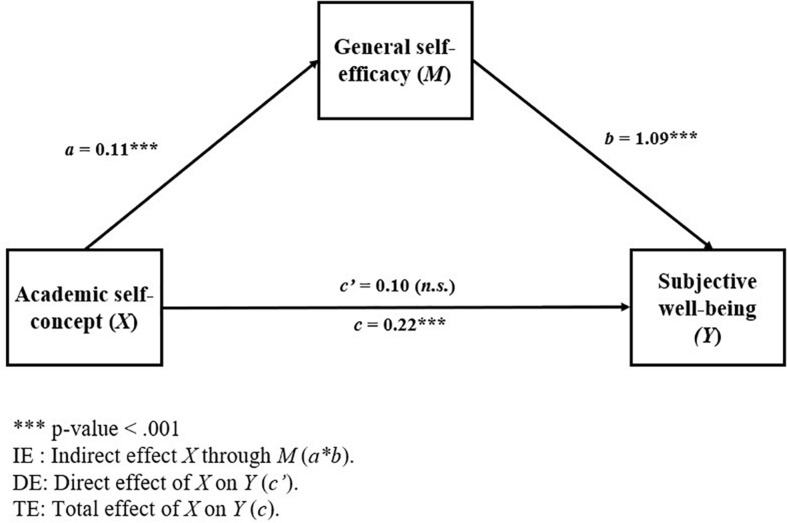
Mediational Model 1: effect of academic self-concept on subjective well-being through general self-efficacy, controlling for gender (subsample: migrants).

On the other hand, the results of the regression analyzes that make up the mediation model 2 are presented in Table 4, while the general diagram of this model is presented in Figure 3.

**TABLE 4:** Linear regression analyzes for Mediational Model 2 (sublample: migrants).

	Consequent							
	
*n* = 179		*M* (General self-efficacy)				*Y* (Subjective well-being)		
				
Antecedent		Coeff.	*SE*	*p*		Coeff.	*SE*	*p*
*X* (Academic self-concept)	*a*	0.11	0.02	< 0.001	*c’*	0.10	0.07	0.16
*M* (General self-efficacy)		−	−	−	*b*	1.10	0.23	< 0.001
Gender		–0.07	0.08	0.39		–0.41	0.25	0.11
Constant	*i*_*M*_	2.29	0.15	< 0.001	*i*_*M*_	3.88	0.69	< 0.001

		*R*^2^ = 0.15				*R*^2^ = 0.19		
		*F*(2,176) = 14.98, *p* < 0.001				*F*(3,175) = 13.29, *p* < 0.001		

A total mediation can be observed in this model, where the total effect (TE: *b* = 0.22, 95% BCa CI [0.09, 0.36]) and the indirect effect (IE: *b* = 0.13, 95% BCa CI [0.07, 0.21]) of academic self-concept on subjective well-being were statistically significant, while the direct effect was not statistically significant (DE: *b* = 0.10, 95% BCa CI [−0.04, 0.24]).

## Discussion

Regarding the study variables, statistically significant differences were found in terms of academic self-concept and general self-efficacy, but not in subjective well-being.

In relation to the highest scores obtained by migrant students in academic self-concept, the results contradict those found in Spain by León del Barco et al. (2007) and Plangger (2015), as well as results obtained in Israel (Ullman and Tatar, 2001) and in Greece (Giavrimis et al., 2003). The explanation for these results could lie in the fact that migrant students come from family environments, where the importance of study as a tool for social mobility has been understood and that in the case of migrant families the situation is exacerbated as a result of arriving in a country in search of opportunities and substantial changes in their socioeconomic situations (Aragonés and Salgado, 2011). The cultural weight that migrant families bring (Shershneva and Basabe, 2012) in terms of intentions of progress and substantial improvements in the quality of life could be generating in their homes a discourse favorable to study and trust in the capacities of adolescents, which would be reflected in that they feel appreciated by their teachers and that they work a lot in class. The fact of feeling good at an academic level is reflected in the grades obtained by migrant students, where self-concept is built in interaction with teachers and classmates who positively reinforce these attitudes, giving as a result a student who feels competent and works to achieve its objectives (Gargallo et al., 2011).

In relation to general self-efficacy, an important contribution of this study is the differences found in the total score of the scale. The results contradict the literature in that migration could show a negative correlation with self-efficacy (Fan and Mak, 1998; Briones et al., 2005; Gómez-Garzón, 2018; Juárez-Centeno, 2018). In this sense, the family environment and somewhat more adverse economic situations could be generating more independent adolescents and with a greater sense of responsibility, affecting their perceptions of the capacities they have to solve problems and the range of possibilities they have to face difficulties. Immigration can bring with it worldviews other than local ones that could be beneficial in terms of innovation, flexibility and other soft skills. This is where transculturation comes into play (González, 2009), since this cultural fusion recovers the best of migrants and natives (Carreón et al., 2016), which could be generating positive changes in the Chilean educational system by enrolling more competive students.

In connection with subjective well-being, studies corried out in other countries by Bilbao et al. (2007), Herrero et al. (2012), Muñoz and Alonso (2016), and Hendriks and Bartram (2019), have found that migrants evidenced lower levels of subjective well-being than their native counterparts. Similar results were even found in Chile by Alfaro et al. (2016). This research exhibited differences favoring natives, however, they were not statiscally significant. These results could be attributed to various factors that are possibly attributable to the fact that the perception of material well-being, health, achievements and future is being managed in a good way by both migrant and native students, as a result of the equal integration of both groups in the health and educational systems with possibilities of personal development through the laws of fee exceptions in higher education and reforms to the system. For example, in terms of satisfaction with health, literature has widely documented the migrants arrive in the host country with better health condition compared with locals, situation that it is likely to be maitained in time (Constant et al., 2018; Luthra et al., 2020). In the specific case of migrant students, they seem to have been successful in restructuring their network of interpersonal relationships in the host country, with no impact on subjective well-being, despite the fact that the literature suggests that the social capital of migrants in general is lower and that this has high correlations with subjective well-being and that it can be a predictor of satisfaction with life (Helliwell, 2003).

Studies such as those by Martínez García et al. (2001) establish a close relationship between subjective well-being and social support, the latter being of great importance in situations of high stress that usually accompany migratory experiences (Berry, 1997). Local governments (municipalities) have made remarkable efforts to integrate migrant students, which seem to have resulted in similar subjective perceptions of well-being among native and migrant students. The absence of statistically significant differences between migrants and natives could suggest that the migration process has not been a traumatic experience for adolescents and that they view their future with optimism.

Regarding gender differences, this study found no statically significant differences in terms of academic self as were also encountered in the contributions by Costa and Tabernero (2012) and the one of Gálvez-Nieto et al. (2017). This may be related to the fact that self-efficacy is related to academic achievement and social adjustment (Trautwein et al., 2006) and both boys and girls would have similar perceptions regarding the achievement of goals. The results obtained are a good incentive since the Government of Chile has highlighted the need to narrow the gender gap in the educational context (Government of Chile, 2013). These results are important because adolescence is a particularly complex period and the literature has reported that it is a more difficult process for girls. Presumably, the results speak of a similar social adjustment in both genders and good socialization, which is especially important given that self-concept is a social product that is generated through the interaction and valuation of others (García and Musitu, 1999). In terms of roles rooted in Latin American societies, male provider and female housewives, it could be expected that girls would show a lower academic self-concept in relation to boys based on expectations. The results do not mirror these assumptions, on the contrary, they could be indications that Chilean society is advancing in terms of equality and equitable treatment in terms of gender and it is also providing a supportive social context. This situation could also be determined by a good attitude of the teachers toward their students regardless of their gender and that the perspectives and aspirations of both girls and boys are not being affected by discrimination and machismo.

Now, the results on self-efficacy mirrored those of Junge and Dretzke (1995), Blanco Vega et al. (2012), and Huang (2013). The differences evidenced could mean the level of achievements and goals will be different in men and that this fact would have repercussions, for example, in the choice and concretion of professional careers and ventures that they decide. Likewise, these results could give indications that the higher levels of resilience in boys could lead to greater well-being in them compared to girls in the future. The fact that boys feel they have more resources to solve unexpected situations and that they feel more confident in their abilities could have repercussions in the world of work and, for example, perpetuate salary gaps by feeling that they “deserve” better positions and salary conditions because they are more self-effective. Society must advance in this regard and promote self-efficacy as an engine in achieving goals and equality.

In terms of subjective well-being, this study found no statistically significant differences regarding gender. These findings are not in line with those of Oyanedel et al. (2015) and Alfaro et al. (2016) in Chile, and in studies in Spain such as that of González-Carrasco et al. (2017). Factors such as material, health, and relations satisfaction could be operating at similar levels in both groups. In line with Casas (1998, 2006), this finding highlights the importance of conducting studies on subjective well-being in adolescent populations in developing countries, contrasting genders in order to advance a conception of well-being beyond meeting basic needs and focusing on the development of adolescent potentials, since happy adolescents are later happy adults. In terms of social support which is highly related with subjective well-being, girls and boys seem to have been successful in structuring their network of interpersonal relationships, exhibiting similar social capital which has high correlations with subjective well-being and that it can be a predictor of satisfaction with life (Helliwell, 2003). In this line, the size of the social network and coverage of basic needs (Basabe et al., 2009) would be positively related to subjective well-being. The results suggest that, at least in this sample, social and structural factors such as access to opportunities, expectations and roles (Stevenson and Wolfers, 2009), which have traditionally favored men in Latin American contexts, may have a window to experience changes that can be reflected in similar subjective well-being indexes for both groups. These changes can also lead to more equal and democratic spaces of study and better opportunities at work places.

As explained, various studies show a positive correlation between subjective well-being and other variables such as self-concept, and self-efficacy (Huebner, 1991; Harter, 1998; McCullough et al., 2000; Luján, 2002; Gutiérrez and Gonçalves, 2013). Literature has also suggested that self-efficacy may mediate the relations between subjective well-being and other constructs such as meaning in life, life satisfaction and self-concept (Krok and Gerymski, 2019). In the case of native-born students, the results controlled by gender assume a direct relationship between academic self-concept and subjective well-being; however, general self-efficacy also presents a mediational role. This partial meditational relation can be explained by the fact that academic self-concept highly predicts subjective well-being in Chilean adolescents; However, academic self-efficacy would exercise a mediational function, since the literature has reported in many studies that higher self-efficacy comes with greater self-concept.

In the case of migrant students, self-efficacy would exercise a total mediational role according to the results obtained. It could be assumed that migrant students came to Chile with a more solid academic self-concept and therefore it would not predict their subjective well-being.

The results obtained could constitute a contribution to the Theory of Achievement Goals outlined a few decades ago (Dweck, 1985; Ames, 1987), which could help us to propose the mediational role of self-efficacy in the relationship between self-concept and subjective well-being. Personal goals, according to Nicholls (1984), would be understood as determining agents of behavior and would therefore be mental representations regarding objectives set in an achievement oriented environment and that determine behavior, affectivity and cognition in different situations, and in the case of migrant students, they face contexts where they put their competences and skills to the test in a setting with new motivations, which Ames (1992) understands as a subjective evaluation of the goal structure that is emphasized in a given situation, in order to achieve social approval and status in a group.

The mediational role of self-efficacy would indicate that a more self-effective individual would show higher levels of subjective well-being, a situation that has been supported by previous evidence (Lachman and Weaver, 1998; Lang and Heckhausen, 2001) and where self-efficacy would turn out to be the greatest predictor of subjective well-being (Halisch and Geppert, 2000; Gómez et al., 2007) in native and migrant students.

In this line, the self-concept of individuals would have the ability to generate relevant changes in their attitudes (Marsh, 2006), so that in terms of achievements it could have effects on subjective well-being, directly in the in the case of native students and via self-efficacy in the case of migrant students.

The results obtained in this study should be taken with the appropriate caution, since more extensive studies and other information-gathering techniques as well as different scales will be required in the future to study in greater depth self-concept, self-efficacy, and subjective well-being and especially the possible causal relationships between these constructs.

New lines of research could emerge from this study, for example the study of the mediational role of self-efficacy with subjective well-being with other constructs such as meaning of life, self-esteem and social support. Among the limitations of this study it can be mentioned that non-random samples and a cross-sectional design were used. The lack of similar studies in the Chilean context to use as a point of reference was also a limitation in some cases.

The new inhabitants of Chile have been looking for quality of life and the importance of studying subjective well-being in adolescents and its influential constructs lies in the possibility of generating inputs for the development of public policies that can arise from the systematic study of the Chilean and migrant population in such a way as to provide key information to relevant actors to make decisions that affect minors in Chile.

## Data Availability Statement

The raw data supporting the conclusions of this article will be made available by the authors, without undue reservation.

## Ethics Statement

The studies involving human participants were reviewed and approved by Comité de Ética de la Facultad de Administración y Economía de la Universidad de Santiago de Chile. Written informed consent to participate in this study was provided by the participants’ legal guardian/next of kin.

## Author Contributions

CC contributed to the conception of the study, involved in planning, supervised the work, processed the experimental data, performed the analysis, interpreted the data, drafted the manuscript, and designed the figures. AR involved in planning and supervising the work, processed the experimental data, performed the analysis, drafted the manuscript, and designed the figures. FV contributed to the conception, analysis, interpretation of data, aided in the sample design, interpreting the results, worked on the manuscript, and revised it critically. SC contributed to the conception, analysis, interpretation of data, aided in the sample design, interpreting the results, worked on the manuscript, and revised it critically. EL-O performed the measurements, sample design, aided in interpreting the results, and worked on the manuscript. JR processed the experimental data, performed the analysis, drafted the manuscript, and designed the figures. All the authors discussed the results and commented on the manuscript.

## Conflict of Interest

The authors declare that the research was conducted in the absence of any commercial or financial relationships that could be construed as a potential conflict of interest.
